# Editorial: The role of leukocyte extracellular traps in Inflammation and autoimmunity

**DOI:** 10.3389/fimmu.2022.1075026

**Published:** 2022-11-01

**Authors:** Daniel Appelgren, Kim Maree O’Sullivan

**Affiliations:** ^1^ Department of Health, Medicine and Caring Sciences, Linköping University, Linköping, Sweden; ^2^ Centre for Inflammatory Diseases, Department of Medicine, Monash University, Melbourne, VIC, Australia

**Keywords:** leukocyte extracellular traps, neutrophil extracellular traps, inflammation, autoimmunity, macrophage extracellular traps, eosinophil extracellular traps, T cell extracellular traps, mast cell extracellular traps

Neutrophil extracellular traps (NETs) are a well-studied form of neutrophil cell death first described by Brinkmann et al. in 2004, which releases extracellular DNA decorated with enzymes, proteases, histones, and reactive oxygen species (1). There is now accumulating evidence that this form of cell death is not unique to neutrophils. It has been discovered that other innate cells such as macrophages (2, 3), mast cells (4), eosinophils (5, 6), and basophils (7) undergo a similar process. It has also become clear that this phenomenon is not restricted to the innate immune system as lymphocytes (both B and T cells), have been found to release extracellular DNA inducing inflammatory responses (8, 9). In this Research Topic we present original and review articles discussing mechanisms of action of leukocyte extracellular traps and their potential as therapeutic targets for inflammatory diseases.

Eosinophil extracellular traps “EETs” where first described in 2008 by Yousefi et al. (5), like NETs they can undergo suicidal EETosis or vital EEtosis (does not result in the death of the cell). In this Research Topic Neves et al. eloquently delineate the ultrastructural features of EETs *in vivo via* transmission electron microscopy. Using tissue samples from eosinophilic diseases (eosinophilic chronic rhinosinusitis, ulcerative colitis and hyper-eosinophilic syndromes in the skin) they show that EETosis results in both granule deposition, eosinophil sombrero vesicles and Charco-Leyden crystals. A unique feature of eosinophils was the observation of fragmentation of the eosinophil nucleus into vesicles with no signs of EETosis. Hashimoto et al. found *in vivo* evidence of EETs in eosinophilic disease by studying tissues sample from patients with eosinophilic granulomatosis with polyangiitis. Cell free nuclear DNA (cf-nDNA) and cell free mitochondrial DNA (cf-mtDNA) were both increased in patient serum and correlated with Birmingham Vasculitis Activity Score (BVAS) scores. *In vitro* investigation of EETs demonstrated that platelets adhered to EET chromatin and that they were more resistant to DNase I treatment than NETs.

Similar to how complexes of histones, proteins and DNA are studies in tissues, this can also be measured in the blood circulation. Matta et al. present a validated method for detection of NETs in patient plasma of patient with SLE. Using the gold standard technique for identification of NETs *in vitro* (H3Cit, MPO and DNA) they designed a multiplex ELISA that reliably detected NETs within plasma samples, with increased sensitivity. They observed increased levels of NETs in SLE patients compared with healthy blood donors only when coating the plate with antibodies against both H3Cit and MPO, but not against only H3Cit. Furthermore, a new smear technique using only 1 µ1 of plasma was introduced to detect NETs (with sytox green to detect extracellular nDNA and MPO, H3Cit and DAPI to stain nDNA). This method produces quantifiable data, which can be analysed using free software (such as Image J).

During the COVID-19 pandemic it became apparent that activation of neutrophils by SARS-CoV-2 resulted in release of NETs that contribute to pathology (10). Detrimental host effects of ETs are often linked to an elevated production and/or a reduced clearance of ETs. Vitkov et al. presents a case where both these aspects are in play and contribute to an increased disease burden in patients with both periodontitis and COVID-19. In the context of periodontitis, the presence of liposaccharide binds to the SARS- CoV-2 S protein and further activates NETs production *via* NF-κb signalling. Scozzi et al. also outline the role of NETs in COVID-19, but focus on the heterogeneous inflammatory condition of acute lung injury. The timing of delivering NET therapeutics is discussed as it is critical - too early in infection can be detrimental to host defence, whereas late delivery can reduce inflammation and reduce pathological damage.

NETs contain numerous known autoantigens which are connected to autoimmune diseases, exemplifying the role NETs play in linking innate and adaptive immunity. Therefore, limiting exposure of NET constituents to the immune system should diminish autoimmunity. Petrelli et al. review the role NETs play in type 1 Diabetes (T1D). Hyperglycemia a clinical feature of T1D, instigates NET formation, with NET remnants found within the pancreas. Furthermore, neutrophils from patients with T1D are phenotypically distinct from those of healthy subjects, with T1D neutrophils displaying interferon signatures similar to what is seen in rheumatoid arthritis and SLE. These atypical neutrophils have reduced capacity to migrate or perform phagocytosis which leads to a compromised host defence in T1D patients. This eloquent review delineates a potential model for the contribution of aberrant neutrophils and NETs over time in T1D.

Lymphocyte extracellular traps were only discovered in 2018/2019 (8, 9). Since then, the knowledge about this mechanism, for at least T cells, have surged and importantly shown to be relevant *in vivo*. Ouyang et al. describe the role of T cell extracellular traps (TETs) within the cutaneous skin environment whereby they contribute to host defence by limiting the spread of pathogenic agents and promote a wound healing environment. Of interest is the role the skin microbiome plays in shaping and regulating T cell ET formation, in the context of skin acne.Th17 cells provide direct antimicrobial activity through the release of ETs. *C. acne* is ensnared within these DNA structures, whereby histones and antimicrobial molecules neutralise the bacteria. Colciaghi et al., discuss the role of ETs contributing to the observed neuroinflammation in several disorders, Alzheimer’s disease, amyotrophic lateral sclerosis, ischemic stroke, traumatic brain injury, spinal injury and brain tumours. The authors have comprehensively outlined how we can therapeutically target the extrusion of extracellular DNA within the CNS, in multiple leukocyte subsets and lymphocytes.

In conclusion this collection of papers highlights the destructive nature of the release of extracellular DNA in multiple pathologies. The common feature of the work within this topic is that there are still large gaps in our knowledge to be filled. Importantly there should be increased impetus to provide therapeutics which specifically target extracellular traps without compromising host defence.

## Author contributions

All authors listed have made a substantial, direct, and intellectual contribution to the work and approved it for publication.

## Funding

KMO is funded by an Australian National Health and Medical Research Council Ideas Grant ID:2001325. DA is funded by the Ingrid Asps Foundation.

## Conflict of interest

The authors declare that the research was conducted in the absence of any commercial or financial relationships that could be construed as a potential conflict of interest.

## Publisher’s note

All claims expressed in this article are solely those of the authors and do not necessarily represent those of their affiliated organizations, or those of the publisher, the editors and the reviewers. Any product that may be evaluated in this article, or claim that may be made by its manufacturer, is not guaranteed or endorsed by the publisher.

## References

[1] Brinkmann V Reichard U Goosmann C Fauler B Uhlemann Y Weiss DS. Neutrophil extracellular traps kill bacteria. Science (2004) 303(5663):1532–5. doi: 10.1126/science.1092385 15001782

[2] King PT Sharma R O’Sullivan K Selemidis S Lim S Radhakrishna N. Nontypeable haemophilus influenzae induces sustained lung oxidative stress and protease expression. PloS One (2015) 10(3):e0120371. doi: 10.1371/journal.pone.0120371 25793977 PMC4368769

[3] Liu P Wu X Liao C Liu X Du J Shi H. Escherichia coli and candida albicans induced macrophage extracellular trap-like structures with limited microbicidal activity. PloS One (2014) 9(2):e90042. doi: 10.1371/journal.pone.0090042 24587206 PMC3934966

[4] von Kockritz-Blickwede M Goldmann O Thulin P Heinemann K Norrby-Teglund A Rohde M. Phagocytosis-independent antimicrobial activity of mast cells by means of extracellular trap formation. Blood (2008) 111(6):3070–80. doi: 10.1182/blood-2007-07-104018 18182576

[5] Yousefi S Gold JA Andina N Lee JJ Kelly AM Kozlowski E. Catapult-like release of mitochondrial DNA by eosinophils contributes to antibacterial defense. Nat Med (2008) 14(9):949–53. doi: 10.1038/nm.1855 18690244

[6] Ueki S Melo RC Ghiran I Spencer LA Dvorak AM Weller PF. Eosinophil extracellular DNA trap cell death mediates lytic release of free secretion-competent eosinophil granules in humans. Blood (2013) 121(11):2074–83. doi: 10.1182/blood-2012-05-432088 PMC3596967 23303825

[7] Yousefi S Morshed M Amini P Stojkov D Simon D von Gunten S. Basophils exhibit antibacterial activity through extracellular trap formation. Allergy (2015) 70(9):1184–8. doi: 10.1111/all.12662 26043360

[8] Ingelsson B Soderberg D Strid T Soderberg A Bergh AC Loitto V. Lymphocytes eject interferogenic mitochondrial DNA webs in response to CpG and non-CpG oligodeoxynucleotides of class c. Proc Natl Acad Sci U.S.A. (2018) 115(3):E478–E87. doi: 10.1073/pnas.1711950115 PMC5776968 29295921

[9] Costanza M Poliani PL Portararo P Cappetti B Musio S Pagani F. DNA Threads released by activated CD4(+) T lymphocytes provide autocrine costimulation. Proc Natl Acad Sci U.S.A. (2019) 116(18):8985–94. doi: 10.1073/pnas.1822013116 PMC6500139 30988194

[10] Veras FP Pontelli MC Silva CM Toller-Kawahisa JE de Lima M Nascimento DC. SARS-CoV-2-triggered neutrophil extracellular traps mediate COVID-19 pathology. J Exp Med (2020) 217(12):e20201129. doi: 10.1084/jem.20201129 32926098 PMC7488868

